# Genomic profiling is predictive of response to cisplatin treatment but not to PI3K inhibition in bladder cancer patient-derived xenografts

**DOI:** 10.18632/oncotarget.13062

**Published:** 2016-11-03

**Authors:** Lei Wei, Sreenivasulu Chintala, Eric Ciamporcero, Swathi Ramakrishnan, May Elbanna, Jianmin Wang, Qiang Hu, Sean T. Glenn, Mitsuko Murakami, Lu Liu, Eduardo Cortes Gomez, Yuchen Sun, Jacob Conroy, Kiersten Marie Miles, Kullappan Malathi, Sudha Ramaiah, Anand Anbarasu, Anna Woloszynska-Read, Candace S. Johnson, Jeffrey Conroy, Song Liu, Carl D. Morrison, Roberto Pili

**Affiliations:** ^1^ Department of Biostatistics & Bioinformatics, Roswell Park Cancer Institute, Buffalo, NY, USA; ^2^ Genitourinary Program, Roswell Park Cancer Institute, Buffalo, NY, USA; ^3^ Department of Cancer Genetics, Roswell Park Cancer Institute, Buffalo, NY, USA; ^4^ Center for Personalized Medicine, Roswell Park Cancer Institute, Buffalo, NY, USA; ^5^ Department of Pharmacology and Therapeutics, Roswell Park Cancer Institute, Buffalo, NY, USA; ^6^ Medical & Biological Computing Laboratory, School of Biosciences and Technology, VIT University, Vellore, Tamil Nadu, INDIA; ^7^ Genitourinary Program, Indiana University-Simon Cancer Center, Indianapolis, IN, USA

**Keywords:** urothelial carcinoma, patient-derived xenograft, PI3KCA

## Abstract

**Purpose:**

Effective systemic therapeutic options are limited for bladder cancer. In this preclinical study we tested whether bladder cancer gene alterations may be predictive of treatment response.

**Experimental design:**

We performed genomic profiling of two bladder cancer patient derived tumor xenografts (PDX). We optimized the exome sequence analysis method to overcome the mouse genome interference.

**Results:**

We identified a number of somatic mutations, mostly shared by the primary tumors and PDX. In particular, BLCAb001, which is less responsive to cisplatin than BLCAb002, carried non-sense mutations in several genes associated with cisplatin resistance, including *MLH1*, *BRCA2*, and *CASP8*. Furthermore, RNA-Seq analysis revealed the overexpression of cisplatin resistance associated genes such as *SLC7A11*, *TLE4*, and *IL1A* in BLCAb001. Two different *PIK3CA* mutations, E542K and E545K, were identified in BLCAb001 and BLCAb002, respectively. Thus, we tested whether the genomic profiling was predictive of response to a dual PI3K/mTOR targeting agent, LY3023414. Despite harboring similar *PIK3CA* mutations, BLCAb001 and BLCAb002 exhibited differential response, both *in vitro* and *in vivo*. Sustained target modulation was observed in the sensitive model BLCAb002 but not in BLCAb001, as well as decreased autophagy. Interestingly, computational modelling of mutant structures and affinity binding to PI3K revealed that E542K mutation was associated with weaker drug binding than E545K.

**Conclusions:**

Our results suggest that the presence of activating *PIK3CA* mutations may not necessarily predict *in vivo* treatment response to PI3K targeted therapies, while specific gene alterations may be predictive for cisplatin response in bladder cancer models and, potentially, in patients as well.

## INTRODUCTION

Bladder cancer, with ~380,000 new cases per year and 15,000 deaths, stands as the ninth most common cancer worldwide. Histologically, more than 90% of the cases are urothelial carcinoma and at time of diagnosis 75-85% of tumors are non-muscle invasive cancer (NMIBC). Approximately 60-70% of NMIBC recurs within one year and 10-20% will progress to muscle invasive disease. Muscle-invasive bladder cancer (MIBC) has the worst prognosis, with a five-year survival rate of less than 50%. Treatment options for MIBC remain cisplatin-based regimen [1]. Therefore, there is a need to develop more clinical relevant models to understand the biology and develop effective therapeutic options for patients with MIBC.

Patient derived tumor xenografts (PDX) have become accepted preclinical models because of their retained original tumor heterogeneity and genetic make-up, suggesting a more reliable drug development tool as compared to tumor cell lines [2, 3]. To date, there is a limited number of established bladder cancer PDX models [4] that are molecularly characterized and available for testing drug resistance and sensitivity. Recent high throughput genomic studies have revealed several gene and pathway alterations associated with MIBC [5, 6], including PI3K/AKT/mTOR and ERK/MEK/RAS pathways as drivers of bladder cancer progression and potential targets for therapeutic interventions [3]. The genomic landscape in MIBC includes alteration of 9 oncogenes and 23 tumor suppressor genes. The reported top five mutated oncogenes are *PIK3CA* (9-20%), *FGFR3* (5-20%), *ERBB3* (11%), *RXRA* (9%), and *ERBB2* (8%) [5]. Among the tumor suppressors, the top 5 gene alterations include *TP53* (24-56%), *MLL* (27%), *ARID1A* (25%), *KDM6A* (24%), and *TSC1* (11-16%) [5]. The genetic characterization mutations reported in bladder cancer have contributed to the molecular subtyping of this disease: *FGFR3* and *TP53* mutations in UroA and UroB cluster [7], *FGFR3* mutation in Cluster I [6], *FGFR3* and *TSC1* mutations in the basal and luminal phenotype [8, 9]. This molecular classification, combined with histopathology analysis, provides the opportunity to develop more effective personalized therapies for bladder cancer patients.

Cisplatin based treatment options have improved the survival in bladder cancer. However, patients eventually develop resistance to treatment and disease progression. Several reports have revealed different potential mechanisms responsible for intrinsic and acquired drug resistance including cisplatin binding, metabolism, transport [10], and intracellular sequestration [11, 12]. As a potential marker for cisplatin resistance, differential expression of GSH synthesis regulating the cystine/glutamate exchanger protein, xCT, has also been reported in bladder cancer [13]. In addition, targeting mTOR pathways in post-cisplatin bladder cancer has been tested, but has not been associated with improved clinical outcome [14]. Accordingly, more clinically and molecularly relevant models are necessary to better understand the molecular alterations associated with drug response, and to develop more effective personalized therapies for MIBC.

In this study, we characterized two PDX tumors recently established in our lab by genomic profiling. As previously reported, BLCAb001 is less cisplatin responsive as compared to BLCAb002 [15], and carries specific cisplatin resistance markers, such as a caspase 8 mutation and over expression of the cystine transporter xCT. Genomic analysis also revealed that both BLCAb001 and BLCAb002 present common *PIK3CA* E542K and E545K driver mutations, respectively. However, the treatment response to the dual PI3K/mTOR inhibitor LY3023414 (LY414) was found to be significantly hampered in BLCAb001, suggesting the presence of alternative pathways. Overall, our data suggest that a comprehensive profiling, rather than solely mutational analysis, may predict response to PI3K/mTOR targeted therapies in bladder cancer.

## RESULTS

### Somatic mutations in primary tumors and PDXs

We recently established two PDXs, BLCAb001 and BLCA002, from two patients undergoing cystectomy for urothelial carcinoma [15]. Based on the previously reported difference in cisplatin sensitivity between the two models, we decided to perform a genomic profiling of the original tumors and the derived PDXs. Using a high-throughput paired-end sequencing approach, we generated 84 to 330 million of 100-bp reads per sample. For non-PDX samples, over 98% of the reads were successfully mapped to the human reference by using BWA. For PDX samples, the mapping rates were 94.5% and 86.6% with human reference. After mapping to the human and mouse combined reference, the mapping rates for these two PDXs increased to 99.1% and 99.2%. All samples reached the designed goal of 80% of the targeted regions covered with at least 30X coverage (Table S1).

**Figure 1:**
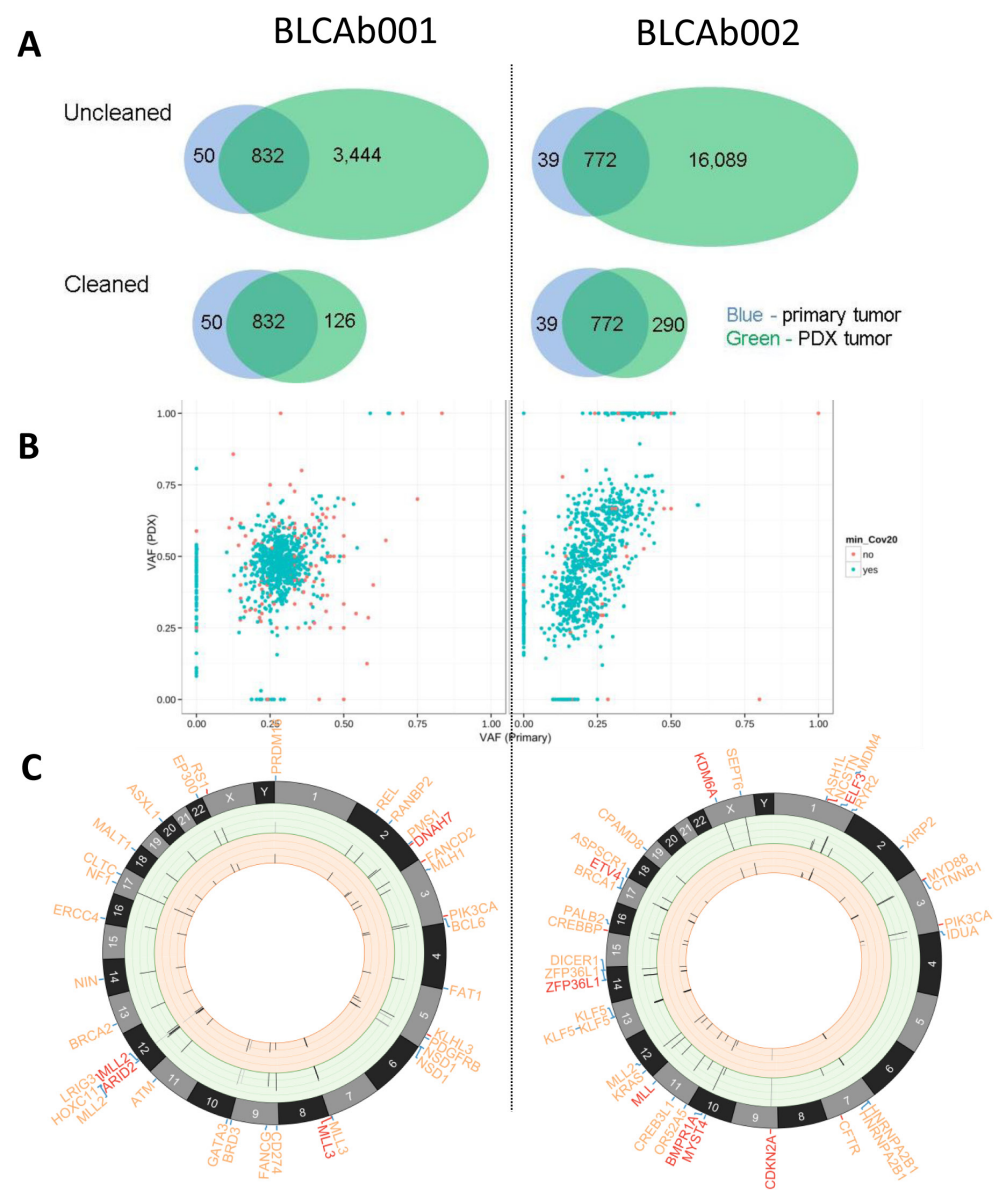
Somatic mutations in primary tumor and PDX **A.** Effects of mouse contamination on somatic mutation calling. Before (“Uncleaned”) and after (“Cleaned”) filtering out mouse contamination, the initial single nucleotide variation (SNV) calls from PDX samples (green) were compared with the matched primary tumor (blue). Top: “Uncleaned”, bottom: “Cleaned”; Left: BLCAb001, right: BLCAb002. The excessive amount of SNV calls in the “Uncleaned” PDX data likely reflects artifacts introduced by mouse contamination. **B.** Plot: variant allele fraction (VAF) defined as the fraction of reads harboring mutant allele for each mutation in the primary tumor and matched PDX. The mutations with less than 20X coverage in primary or PDX tumor are highlighted in red. **C.** Circos plots [61] depicting nonsynonymous genetic alterations that were: 1) previously reported by ClinVar or COSMIC or 2) novel variant in a Cancer Gene Census gene or other genes known to be recurrently mutated in bladder cancer. These two categories are distinguished by the color of connection between gene symbol and chromosome: red = 1), blue = 2). From outer to inner components: gene symbol (red, truncating mutations including nonsense and splice-site SNV, frameshift Indel; orange, alternating mutations including missense SNV and in-frame Indel), chromosomes, variant allele frequency (VAF) bars for the corresponding point mutation (range = [0:1], the color of VAF stick indicate coverage: light grey = 0-9X; grey:10-29X; black: > = 30X) in PDX (ring background = green) and the primary tumor (ring background = orange).

Filtering out mouse contamination was a critical step in order to obtain accurate mutation calls in the PDX samples. In a test run on the unfiltered data, we identified 4,276 and 16,861 SNVs in BLCAb001 and BLCAb002, respectively (Figure 1A). The majority of these SNVs was not identified in the primary tumor and was likely caused by mouse contamination. After filtering out mouse reads, most of these suspicious mutation calls disappeared and the remaining mutations were highly consistent with the matched primary tumor. For BLCAb001, we identified 1,008 SNVs and 5 Indels from the primary and PDX and 1,101 SNVs and 14 Indels from BLCAb002. The identified mutations were then manually reviewed to ensure accuracy. After manual review, there were 919 mutations (917 SNVs and 2 Indels) left in BLCAb001 and 980 mutations (973 SNVs and 7 Indels) left in BLCAb002.

The mutation profiles were compared between the primary and PDX to determine similarity. In both cases, majority (91% in BLCAb001 and 82% in BLCAb002) of all mutations were shared by primary and PDX samples (Figure 1B). There also existed smaller numbers of sample-specific mutations (2% primary-unique and 7% PDX-unique in BLCAb001, 3% primary-unique and 15% PDX-unique in BLCAb002), which may reflect the tumor progression from primary to PDX tumors. In BLCAb001, the VAFs of the shared mutations were centered near primary = 0.25 and PDX = 0.5, which may indicate higher tumor purity in PDX than the primary tumor. Similarly, in BLCAb002, most shared mutations were around primary = 0.25 and PDX = 0.5. Additionally, there was a small group of mutations near primary = 0.4 and PDX = 1.0, which were likely to be homozygous in the PDX (Figure 1B).

Among the identified somatic mutations, 13 alterations were previously found clinically relevant according to ClinVar or mutated in other cancers as summarized by COSMIC, including RS1 R209H, PIK3CA E542K, MLL3 R199*, LRIG3 E576K, KLHL3 S410L, FANCD2 L1134V, DNAH7 R1957* (BLCAb001); and PIK3CA E545K, NCSTN S389C, MYD88 S219C, CREBBP W1472C, CFTR R1066C, CDKN2A E69*(BLCAb002) (Figure 1C). Most of these mutations were present in both primary and PDXs except for *CFTR R1066C*, which was only present in the PDX. For other novel nonsynonymous mutations, 57 mutations occurred in Cancer Gene Census genes or other genes known to be recurrently mutated in bladder cancer genes [6, 36, 37], including 10 predicted loss-of-function mutations (BLCAb001: MLL2 Q1361*, ARID2 L47fs; BLCAb002: ZFP36L1 F253fs, MYST4 E1398fs, MLL S2663*, KRAS E3_splice, KDM6A Q958*, ETV4 Q170*, ELF3 D223fs, BMPR1A R361*). The majority (55/57) of these potentially important mutations were present in both the primary and PDX. Only two mutations, *RANBP2 P1380R* in BLCAb001, and *RYR2 E1859K* in BLCAb002, were present in the PDXs but not in the primary tumor (Table S2).

### Mutation signature in 3 groups: common, primary unique, and PDX unique

All SNVs in one patient were segregated by their presence status in primary and PDX tumors into three groups: “Common”, “Unique to primary” and “Unique to PDX”. We analyzed mutation signature in every group. In all three groups in BLCAb001 and two groups (“Common” and “Unique to primary”) in BLCAb002, the mutation signatures were dominated by C > T transitions and C > G transversions. However, in BLCAb002 “Unique to PDX”, the two most prevalent patterns were C > T transition and C > A transversions. Additionally, this group also had elevated T > A transversions, which were not observed in any other groups (Table S2).

### Basal and luminal phenotype in BLCAb001 and BLCAb002, respectively

**Figure 2:**
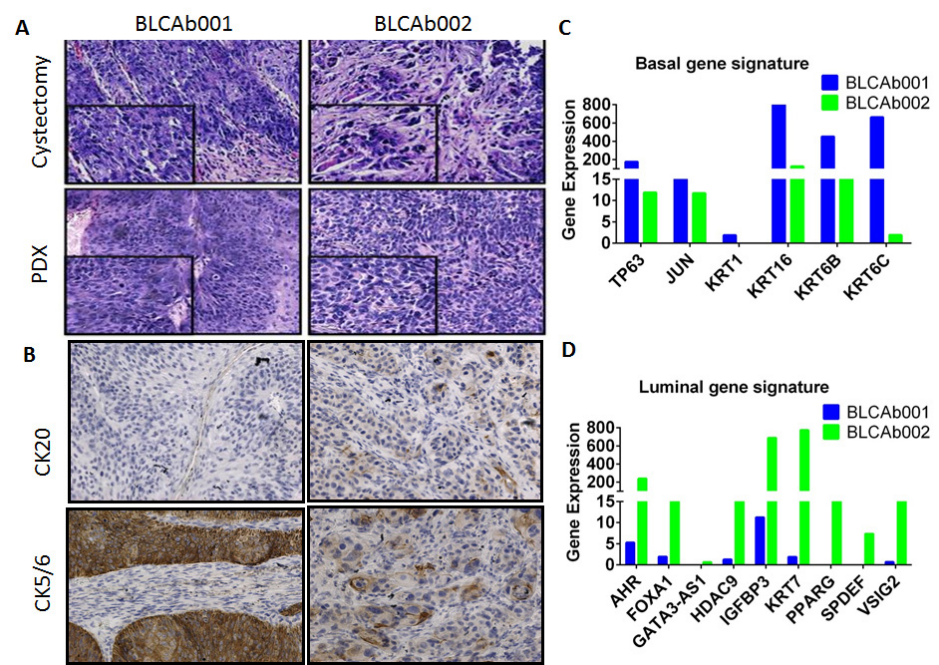
Histological and molecular representation of BLCAb001 and BLCAb002 **A.** H&E staining of original cystectomy and PDX tumors. **B.** Expression of cytokeratine 20 (CK20), CK5/6 in BLCAb001 and BLCAb002. **C.** RNAseq analysis of luminal and basal phenotype gene signature in BLCAb001 and BLCAb002.

BLCAb001 and BLCAb002 were established from cystectomy specimens and maintained the histological features of the original tumors (Figure 2A). Thus, we decided to better characterize the histological phenotypes. Immunohistochemical analysis revealed that BLCAb001 expresses lower levels of the tissue differentiation marker cytokeratin 20 (CK20) (Figure 2B), while had higher expression of cytokeratin CK5/6, as compared to BLCAb002. RNA-Seq analysis confirmed the upregulation of basal phenotype associated genes in BLCAb001 (Figure 2C) and the upregulation of luminal phenotype associated genes in BLCAb002 (Figure 2D).

### Alteration of cisplatin resistance associated genes in primary tumors and PDXs

**Figure 3:**
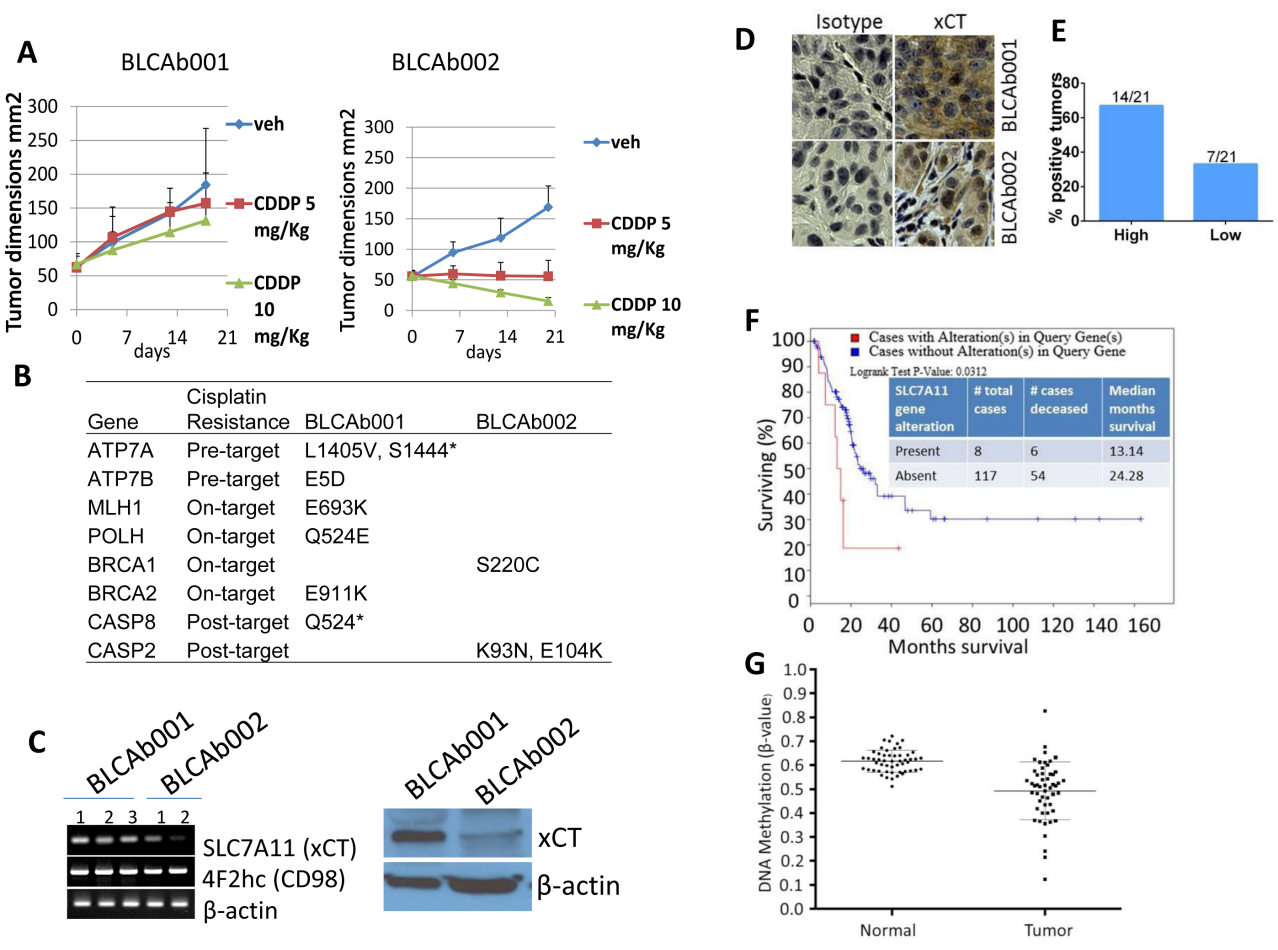
Differential response to cisplatin treatment in PDXs and associated mutational status and gene expression **A.** Cisplatin treatment (5 mg/kg and 10 mg/kg) had a differential effect on BLCAb001 and BLCAb002 *in vivo* growth. **B.** Mutational status of cisplatin resistance associated genes in BLCAb001 and BLCAb002. **C.** RT-PCR and Western blot analysis of *SLC7A11* (xCT), a cisplatin resistance associated gene in BLCAb001 and BLCAb002 PDXs. **D.** Immunohistochemical evaluation of xCT in BLCAb001 and BLCAb002. **E.** Percent tumors with high and low xCT expression. Immunoscore determined as high (80-100% cells positive) and low (2-20% cells positive) [35]. **F.** TCGA data analysis of cBioportal showing the poor survival of patients with alteration (upregulation) of *SLC7A11* (xCT). Logrank test p-value 0.0312. **G.** *SLC7A11* gene methylation in human bladder cancer patients primary tumors (*n* = 22) and the matched non-tumor tissues (*n* = 106). This graph shows the presence of DNA hypomethylation within the *SLC7A11* locus. Each data point represents the average methylation of 2 CG sites most significantly hypomethylated in tumors when compared to normal bladder tissues.

BLCAb001 and BLCAb002 had similar mutation burden, but exhibited a different response to increased doses of cisplatin (Figure 3A). We confirmed this differential response in primary tumor cells isolated from these tumors. Cells isolated from BLCAb001 were found to be less responsive to cisplatin treatment than BLCAb002 cells [15]. To determine whether specific genomic alterations were responsible for the observed different cisplatin sensitivity between BLCAb001 and BLCAb002, we compared the somatically genomic alterations in known cisplatin resistance associated genes [38]. Eight of such genes were found to be mutated (Figure 3B), six in BLCAb001 and two in BLCAb002. Two of these mutations were predicted to cause loss-of-function: ATP7A S1444* and CASP8 Q524*. All other mutations were missense SNVs. In support of a genomic signature associated with cisplatin resistance, RNA-Seq analysis revealed overexpression of known cisplatin resistance associated genes in BLCAb001, including *NRG1, EGFR, SLC7A11, TLE4*, and *IL1A* (Figure S2) [39].

### Overexpression of xCT is associated with cisplatin resistance

Differential response to cisplatin treatment has also been associated with overexpression of the cystine transporter xCT (encoded product of *SLC7A11*) [10, 13]. Interestingly, BLCAb001 showed higher gene and protein expression of xCT, as compared to BLCAb002 (Figure 3C). RNA-Seq analysis confirmed the overexpression of *SLC7A11* in BLCAb001 (Figure S2). Further, immunohistochemical evaluation also showed high expression of xCT in BLCAb001 (Figure 3D). Additionally, we evaluated xCT expression in bladder cancer tumors (*n* = 21) arranged in a tissue microarray (TMA) by immunohistochemistry. High expression of xCT (Figure 3E) was found in 67 % (14 out of 21) of tumors, including PDX BLCAb001. TCGA data analysis for xCT expression revealed the poorer survival of bladder cancer patients is associated with altered expression (Figure 3F). These results suggest that patients with urothelial cancer showing high expression of xCT may have shorter survival due to reduced response to cisplatin-based regimens. Interestingly, a recent study revealed the epigenetic alteration of microRNA-27A leading to xCT overexpression in bladder cancer [13]. To date, the effect of gene methylation on the expression of xCT has not been investigated. In order to correlate xCT expression and methylation, we determined the *SLC7A11* (xCT) methylation status in bladder cancer patients (*n* = 52) and their matched non-tumor tissues (*n* = 106) and found significant hypomethylation of *SLC7A11* in tumors compared to their matched non-tumor tissues (Figure 3G).

To evaluate the association between xCT overexpression and cisplatin resistance, human bladder cancer T24 cisplatin resistance cells were generated upon *in vitro* long drug exposure. As shown in Figure S4A, cisplatin resistant T24 cells presented a > 1-fold increase in IC50 as compared to the parental cells. The decreased sensitivity to cisplatin was associated with an increase in xCT expression (Figure S4B). CD44 expression has been reported to be involved in cisplatin resistance [40] and its isoform CD44V6 has a potential role in stabilization of xCT [41]. Thus, we investigated the expression of CD44 standard (CD44s) and its variants, CD44v6 and CD44v8, in the T24 and UMUC3 models. By q-PCR we observed that, while there was no difference in CD44s and CD44v8 gene expression between parental and cisplatin-resistant cell lines, there was a CD44v6 overexpression in cisplatin resistant T24 and UMUC3 cells as compared to the parental cells, confirming a possible role for xCT (Figure S4C). Next, we tested whether the use of a putative xCT inhibitor, sulfasalazine (SASP), was able to affect the response to cisplatin. Combination of SASP enhanced response to cisplatin in both cisplatin sensitive and cisplatin resistant cells. (Figure S4E and S4F). In addition, SASP effectively inhibited the colony formation of the cisplatin resistant cells, suggesting a role for lysosome function/biogenesis in the survival of xCT overexpressing T24 cells (Figure S4F). In addition, we performed our proliferation assay using the special RPMI media without cysteine, methionine, and glutamate. Supplementation of cysteine, but not methionine or glutamate, to the medium rescued the normal growth of T24 cells (Figures S5A and S5B). Taken together, these results suggest a potential role for targeting the cystine transporter to modulate cisplatin sensitivity in bladder cancer.

### *PI3KCA* mutation status does not correlate with response to a PI3K/mTOR dual inhibitor

**Figure 4:**
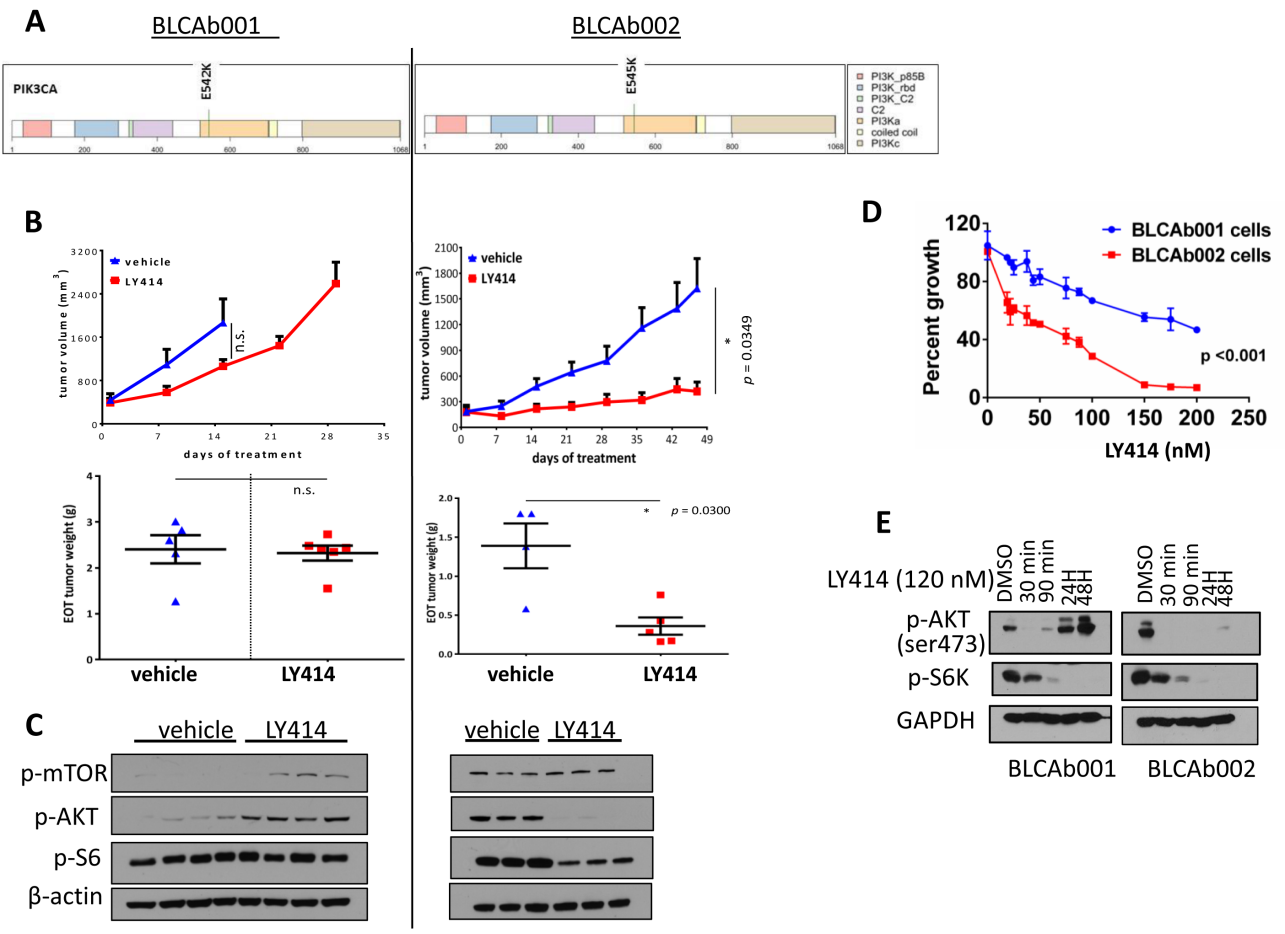
Differential response of *PIK3CA* mutated BLCAb001 and BLCAb002 to LY414 **A.** PI3KCA mutations in BLCAb001 (E452K) and BLCAb002 (E545K). Color codes- Yellow orange color indicates P13K alpha helical domain. **B.** Anti-tumor effects of LY414 on PDXs BLCAb001 and BLCAb002 tumors (upper panels), tumor weights at the end of treatment (EOT) (lower panels). **C.**. Western blot analysis of the effect of LY414 treatment on p-AKT, p-mTOR, and pS6 in BLCAb001 and BLCAb002 PDXs. **D.** Effect of LY414 on BLCAb001 and BLCAb002 cells isolated from PDX cultured in DMEM. Statistical analysis of ANOVA was performed to determine the significance p≤ 0.001. **E.** Western blot analysis of the effect of LY414 treatment on p-AKT and p-S6K in BLCAb001 and BLCAb002 derived cells cultured in DMEM.

The RNA-Seq analysis of BLCAb001 and BLCAb002 revealed that both patients harbored a *PIK3CA* hotspot mutation but on different residues: E542K and E545K in BLCAb001 and BLCAb002, respectively (Figure 4A). These mutations were present in the original tumors and in the established PDXs, and were confirmed in the derived cells lines (Table S2). Since *PI3KCA* helical hotspot mutations (E542K and E545K) are common (25%) in bladder cancers [42], we were interested to test whether this mutational status was associated to response to a targeted therapy. *In vivo* treatment with the dual PI3K/mTOR inhibitor LY3023414 (LY414) demonstrated a significant anti-tumor effect only in BLCAb002 and not in BLCAb001 (Figure 4A and 4B). The inhibition of tumor growth by LY414 was associated with inhibition of phosphorylated AKT (ser473) and phosphorylated S6 kinase (evaluated after 24h of drug treatment) in BLCAb002 but not in BLCAb001 (Figure 4C). While the initial tumor size is about 20% lower in BLCAb002 tumors compared to BLCAb001 tumors, the final size of the tumor at the end of treatment is about 3 fold (150%) lower in BLCAb002 tumors. We found there was no tumor size difference with the treatment in BLCAb001 tumors. We have repeated the experiment with same initial tumor size of BLCAb001 and BLCAb002 tumors and found significant tumor growth inhibition in BLACb002 tumors but not in BLCAb001 tumors (Figure S10). These results suggest that some additional molecular alterations or the cross talk of cisplatin resistant genes may contribute to the relative resistance of BLCAb001 to LY414 despite the presence of an activating *PI3KCA* mutation. Indeed, our RNAseq analysis revealed the overexpression of known genes associated with resistance to AKT inhibition such as phosphatidylinositol-4-phosphate 3-kinase C2 domain-containing gamma polypeptide (*PIK3C2G*), insulin receptor substrate 1 (*IRS1*) and serum- and glucocorticoid-regulated kinase (*SGK1*) (Figure S6) [43-45].

Thus, we evaluated the cytotoxic effects of LY414 on cells isolated from the two PDXs. Similarly to the *in vivo* results, BLCAb002 cells were found to be more sensitive to LY414 as compared to BLCAb001 (Figure 4D). The IC50 for LY414 was 143.94 nM and 45.06 nM in BLCAb001 and BLCAb002 cells, respectively. Interestingly, the target of LY414 p-AKT (ser473) was inhibited in both cells at an early time point, but only in BLCAb002 at 24 and 48 hrs (Figure 4E). Same results were observed for thr308 phosphorylation site (Figure S8A). We observed p-S6 inhibition in both the tumor cell models, also at later time points. When we tested selected agent for either mTOR (RAD001) or AKT (MK2206) inhibition, we observed target inhibition in both BLCAb001 and BLCAb002 (Figure S8B). The inhibition was maintained also at 24 and 48 hrs (Figure S8C). In order to confirm that BLCAB001 tumors in general are less sensitive to the drugs targeting P13K/mTOR inhibitors compared to BLCAb002 tumors, we have determined the p-AKT, downstream makers after treating the cells with BEZ235 (250 nM) and BKM120 (500nM). As the results shown with LY414 treatment, we found pronounced inhibition of p-AKT in BLCAb002 cells which are more sensitive to P13K/mTOR inhibitors compared to BLCAb001 cells (Figure 9SA, 9SB). Interestingly, when we combined LY414 with bromodomain inhibitor JQ, we observed inhibition of p-AKT in BLCAb001 cells, which was not observed with LY414 alone (Figure 9S C). Additional studies are warranted to investigate how JQ combination optimized the P13K/mTOR dual inhibitor LY414.

**Figure 5:**
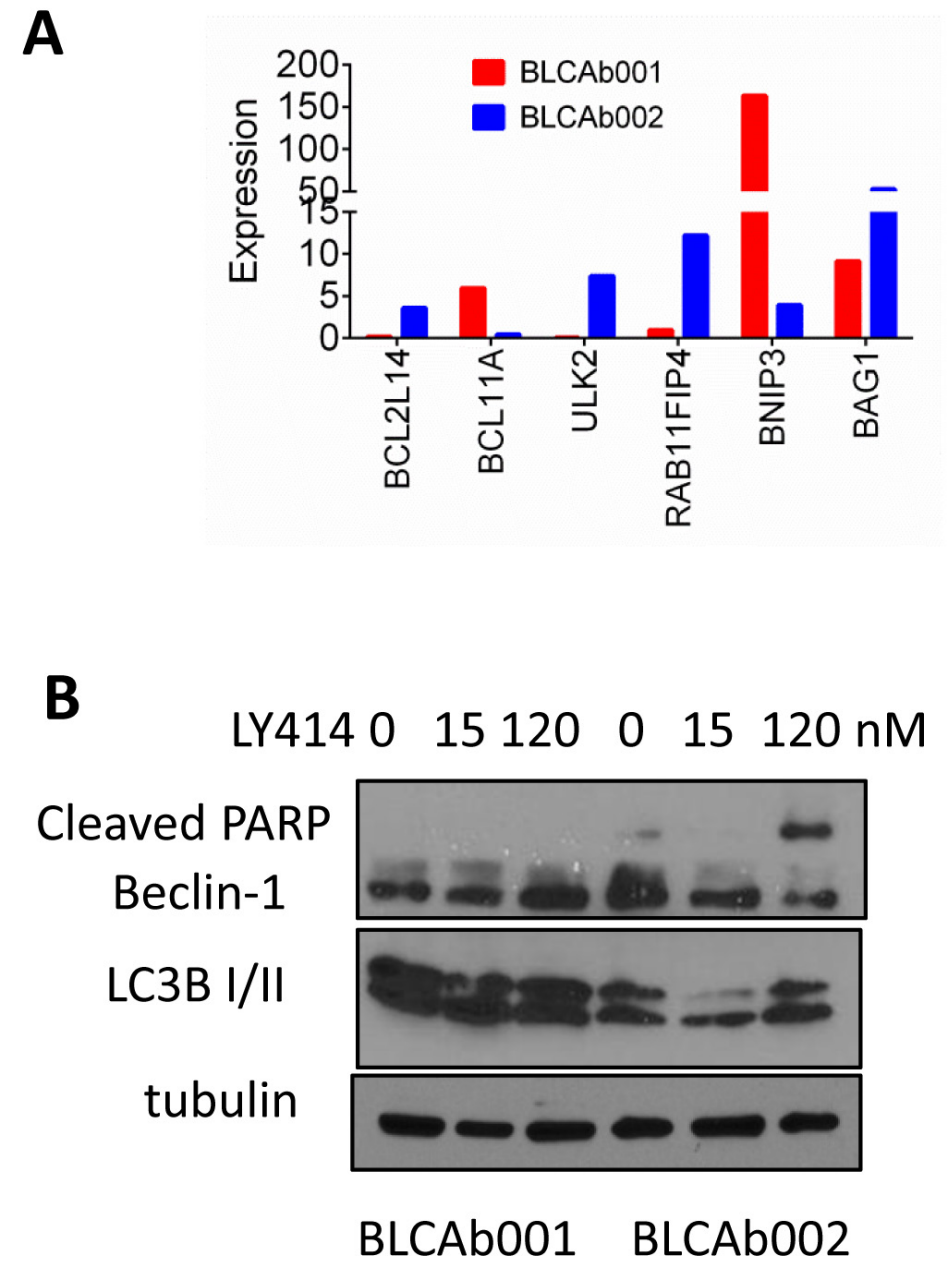
Differential expression and modulation of autophagy in BLCAb001 and BLCAb002 **A.** RNA-Seq analysis data showing the differential expression of autophagy genes in BLCAb001 and BLCAb002. **B.** Differential effect of LY414 on autophagy and apoptosis markers in BLCAb001 and BLCAb002 derived cells.

Interestingly, the differential cytotoxicity effect of LY414 between the two models was not observed when the cells were tested in insulin enriched RPMI F media (Figure S7A). The IC50 was 212.9 nM and 208 nM for BLCA001 and BLCAb002, respectively (Figure S7B). These results suggest that enriched media with insulin may compensate the AKT pathway inhibition. Finally, we examined whether modulation of autophagy could be responsible for the observed differential response to LY414 between the two models. Thus, in our RNA-Seq analysis we observed overexpression of *BNIP3* and *BCL11A*, and downregulation of *BCL2L14*, *ULK2*, *RAB11FIP4* and *BAG1* in BLCAb001 tumors as compared to BLCAb002 (Figure 5A). Western blot analysis confirmed the persistence of autophagy protein expression in BLCAb001 cells treated with LY414, but downregulation of Beclin-1 and LC3B I/II in BLCAb002 cells, associated with cleaved PARP.

### E542 mutation is associated with weaker binding of LY414 to PI3K

**Figure 6:**
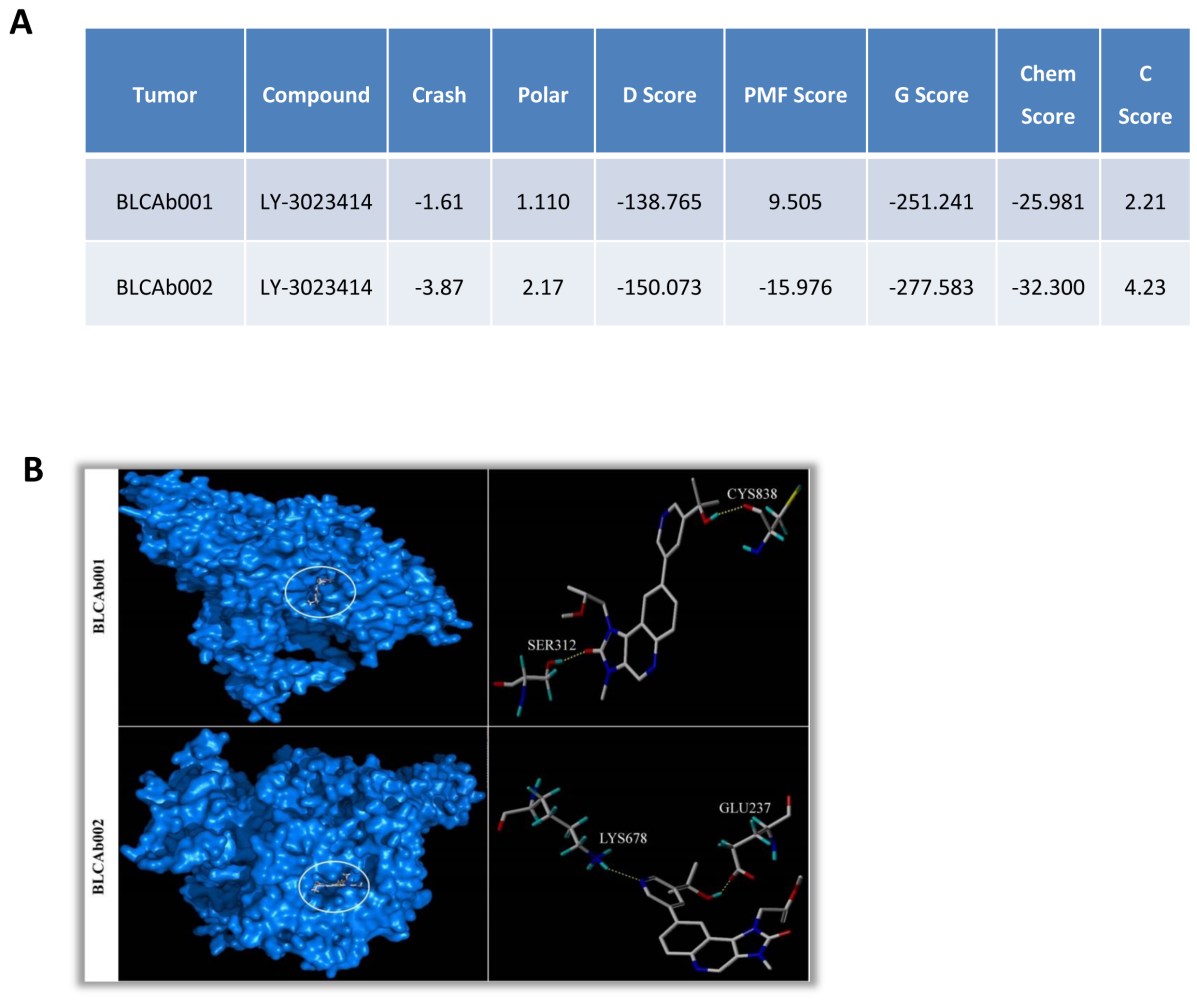
Effect of E542K and E545K mutations on LY414 binding to PI3K **A.** Docking results for BLCAb001 and BLCAb002 with LY414 (Kcal/mol). The Crash score reveals inappropriate penetration into the binding site. The Polar score identifies the Region of the ligand. The D score defines the charge and van der waals interactions between the protein and ligand. The PMF score defines the Helmholtz free energies for protein-ligand atom pairs interactions. The Gscore identifies the Hydrogen bonding, ligand-protein and internal ligand-ligand energies. The Chem score identifies the points for hydrogen bonding, lipophilic contact and rotational entropy, along with an intercept term. Finally, the C-score is the consensus scoring which uses multiple types of scoring functions to rank the overall affinity of ligands. A higher C-score value denotes a greater binding affinity. The increased negative values of Crash score, Chem score, D-score, G-score and Potential Mean Force scores (PMF) indicate the high binding energy between the protein-ligand complexes. The low values indicate the least binding affinity of the compound towards the target. The binding energies are expressed in Kcal/mol unit. **B.** Binding mode in BLCAb001 (E542K mutation) and BLCAb002 (E545K mutation).

Based on the different biological effect elicited by LY414, we investigated whether the mutation type could contribute by affecting the affinity of the compound to the substrate. Protein-ligand binding orientation was analyzed with the Sybyl-X 2.0 program. After docking, the best binding conformation of the compound with the protein was selected based on C score values. C score is the scoring system used to rank the binding affinity of ligands and is based on different scoring functions, including crash score, polar score, D score, PMF score, G score and Chem score, ranging between 0 (low) to 5 (high). The binding parameters for BLCAb001 and LY-3023414 are presented in Figure 6A. The BLCAb001 and LY-3023414 binding had a C-score value of 2.21. The LY-3023414 interacts with residues Ser 312 and Cys 838 as depicted in Figure 6B. BLCAb002 and LY-3023414 complex has a C-score value of 4.23 and it interacts with Glu 237 and Lys 678 residues of BLCAb002 (Figure 6B). The binding of LY-414 to the wild type protein had a C score of 2.51 which is similar as BLCAb001 (data not shown). These results suggest that the E545K mutation observed in BLCAb002 may induce conformational changes in the PI3K protein that could lead to greater binding of LY414 and a stronger inhibitory effect. Lysine is a very dynamic amino acid in the proteins, being oxidized by lysyl oxidase (LOX) an enzyme whose functional significance on tumor progression and metastasis is known [46, 47]. Further, oxidation of specific position lysine molecule opens the protein-protein interactions that influence the tissue remodeling and drug response. Since the E545 mutation we found in BLCAb002 has a lysine at 545 position that may be involved in formation of drug binding pocket and more LY414 drug may bind to PI3K and inhibit the protein.

## DISCUSSION

Precision medicine aimed to develop more tailored and effective cancer treatments is currently based on genomic profiling of either tumor tissues or liquid biopsies. The clinical challenge is whether the molecular findings are predictive of response to targeted therapies in cancer patients. Our preclinical observations suggest that, at least in bladder cancer, a composite genetic signature may be more predictive of response to cisplatin than a specific mutational status to a PI3K inhibitor.

Patient-derived xenografts (PDXs), along with genetically engineered mouse models (GEMMs), are becoming an important tool for drug development in several tumor types, including urothelial carcinoma [48]. In our exosome sequencing study, the PDXs maintained the same genetic make-up as the original tumors. In each case, 80-90% of all somatic mutations were present in both the primary and PDX tumors. There was only a small fraction (7% and 15% in BLCAb001 and BLCAb002, respectively) of PDX-specific mutations, and even a smaller number (2% and 3% in BLCAb001 and BLCAb002, respectively) of mutations present only in the primary tumor. These small differences may reflect either the addition or loss of mutations during tumor progression from primary to PDXs. Alternatively, some of these genetic differences may be explained by tumor heterogeneity. Overall, our results in bladder cancer models confirm the genomic similarity between the original tumor and the derived PDXs as reported also in other tumor types, and support the clinical relevance of utilizing bladder cancer PDXs for drug development.

Cisplatin resistance is one of the major challenges for the management of bladder cancer. The resistance mechanisms may be intrinsic or acquired [5, 49]. Several mechanisms have been proposed, including reduction of intracellular drug accumulation by either reduced uptake [38] or increased efflux by transmembrane pumps, such as the multidrug resistance 1 (MDR1)/p-glycoprotein and ATP binding cassette (ABC) protein [49]. Furthermore, overexpression of MDR1 has been shown to enhance DNA damage repair and to reduce induction of apoptosis [50]. In addition, solute carrier importers CTR1, the SLCs, AQP2, AQP9 and endocytic recycling compartment have been reported as cisplatin resistance regulators [51]. Upregulation of novel genes, including *LUM, DCN, PDE3B, PDGF-C, NRG1*, *PKD2, IL1A* have been shown to be overexpressed in cisplatin resistant cell lines [39]. In our cisplatin resistant PDX model, BLCAb001, we observed genomic alterations of several cisplatin resistance associated genes, such as *Casp8, SLC7A11, TLE4*, and *IL1A*. We have also observed overexpression of the membrane bound cystine/glutamate exchanger xCT, a lysosome regulatory protein and its stabilizer CD44v6 in cisplatin resistant PDX and cells. Recent studies have reported the association of the cystine transporter xCT with cisplatin resistance in bladder cancer [13], and its potential prognostic role in hepatocellular carcinoma [52]. Drug repurposing of sulfasalazine, a xCT inhibitor, is currently in clinical testing [53]. Taken together, our findings suggest a role for xCT in the resistant phenotype and provide not only an additional potential predictive marker to be incorporated in a composite molecular signature for cisplatin resistance, but also a rational target for therapeutic interventions in patients with bladder cancer.

Genomic profiling of our PDX models showed two canonical *PIK3CA* mutations, which activate the PI3K/AKT/mTOR signaling pathway. BLCAb001 possesses a *PIK3CA* activating mutation (E542K), which is less common than E545K present in BLCAb002 [54, 55]). Thus, we expected both tumors to respond to a targeted PI3K inhibitor [56, 57]). To our surprise, LY414 treatment did not significantly inhibit either the *in vitro* or *in vivo* growth of BLCAb001, and did not induce sustained target modulation, as compared to the BLCAb002 model. Several potential mechanisms may be responsible for this differential response. Our RNA-Seq data analysis revealed overexpression of several genes, including *SGK1*, *IRS1*, and *PIK3C2G* in BLCAb001 (Figure S6), which are known resistance markers for P13K/AKT targeted agents [43-45]. Interestingly, when we cultured BLCAb001 cells in enriched RPMI F medium with growth factors and insulin we were able, in part, to restore sensitivity to LY414 (Figure S7). Thus, we speculate that the *in vitro* presence of insulin may artificially hyper-activate PI3K signaling in BLCAb001 cells that is responsible for increased glycolysis and aldolase mobilization leading to increased vulnerability [58]. As shown in the *in vitro* experiments, we observed LY414 induced inhibition of p-AKT, both at the ser473 and the thr308 phosphorylation sites, in BLCAb001 only at early time of exposure (30-90 minutes), but not at 24 and 48 hrs. If we used a selected AKT inhibitor we did not observed this difference. Thus, we hypothesize that in BLCAb001, but not in BLCAb002, inhibition of PI3K may induce a feed-back upregulation of receptor tyrosine kinases and, consequently, re-induction of pAKT and sustained autophagy, as reported in other tumor systems [59]. Recent studies have shown activation of autophagy as one of the critical molecular alterations that limits the anti-tumor effects of PI3K/mTOR inhibitors [60]. Our RNAseq data showed *BNIP3* and *BCL11A* upregulation, and *ULK2, BAG1, RAB11FIP4* and *BCLL14* downregulation in BLCAb001 tumors, mirroring the higher levels of LC3B I/II by Western blot analysis (Figure 5A and 5B). Interestingly, the treatment of sulfasalazine, an inducer of autophagic cell death, enhanced the efficacy of cisplatin resistant BLCAb001 cells. Taken together, these observations suggest that the autophagy may contribute to the observed resistance LY414 in BLCAb001 cells.

The presence of two similar but distinct hot-spot mutations in BLCAb001 and BLCAb002 lead us to explore the possibility that the differential mutational status could have an impact on the affinity for LY414. Interestingly, the docking results suggest that there might be a significant difference in the binding of the compound with the two mutants. Simulation of BLCAb002 and LY414 interaction suggest a higher binding affinity than the BLCAb001 and LY414 complex, as indicated by the differences in C scores. Thus, when we compared the binding affinity of LY414 with the mutants (BLCAb001 and BLCAb002) and the wild type, the best binding affinity for the compound was observed for BLCAb002. This observation may provide an additional mechanism responsible for the differential effect of LY414 in the two bladder cancer PDXs and raises the question whether different mutations may have different drug binding affinities and, consequently, different drug sensitivity. We can speculate that the difference in binding affinity due to conformational changes may be also in part responsible for the lack of sustained *in vitro* target modulation by LY414 observed in BLCAb001. To our knowledge, there are no prior reports showing potential differential effects of E542K and E545K mutations on PI3K drug binding. These findings might provide a rationale for focusing the development of LY414 specifically for bladder cancer patients with a E545K mutation. Additional experimental validation studies are required to confirm these findings.

In conclusion, our results suggest that the identification of *PIK3CA* mutations through genomic profiling may not necessarily predict response to PI3K targeted therapies in mouse models and, likewise, in patients with urothelial carcinoma. Several “by-pass” mechanisms may influence treatment response. In our PDX models, we observed that a composite genetic and molecular signature may be more likely associated with cisplatin sensitivity/response. Additional molecular studies are warranted to identify specific gene mutations/alterations responsible for the differential response to treatment in these PDX models. The development and validation of genomic signatures will help the clinical implementation of effective targeted therapies for bladder cancer.

## MATERIALS AND METHODS

The RPCI Institutional Review Board gave approval for this study. Patients consented to remnant tissue procurement for next generation sequencing of their samples. Sequencing was performed according to a RPCI IRB approved investigator initiated protocol.

### Whole exome sequencing (WES)

Individual exome capture of each DNA sample was carried out using the SureSelectXT Reagent kit as per manufacturer's recommendation (Agilent Technologies, Inc.). 3 μg of genomic DNA was fragmented to a size range of 150-200 bp followed by end repair, adaptor ligation, and low cycle (5) PCR. Libraries were purified and validated for appropriate size (225-275 bp) on a 2100 Bioanalyzer High Sensitivity DNA chip (Agilent Technologies, Inc.). 750 ng of purified library was then hybridized to the SureSelectXT Human All Exon 50 Mb library for 18 hours at 65°C. The captured regions were then bound to Streptavidin T1 magnetic beads (Life Technologies, Inc.) and washed to remove any non-specific bound products. Eluted library underwent a second 11 cycle PCR amplification using Herculase II Fusion Polymerase (Agilent Technologies, Inc.) to add sample specific barcodes necessary for multiplexing. Final libraries were purified, validated for size by a BioAnalyzer (250-350 bp), and quantitated using KAPA qPCR. Individual libraries were pooled (3-plex) in equimolar 2 nM final concentration. Each pool was normalized to 10 pM, loaded and clustered to individual lanes of a HiSeq Flow Cell using an Illumina cBot (TruSeq PE Cluster Kit v3), followed by 2 × 101 PE sequencing on a HiSeq2000 sequencer according to the manufacturer's recommended protocol (Illumina Inc.).

### Detection of somatic sequence mutations in primary tumor

High quality paired-end reads passing Illumina RTA filter were aligned to the NCBI human reference genome (GRCh37) using BWA [16]. PCR duplicated reads were marked using Picard [17]. Putative SNVs and Indels were identified by running variation detection module of Bambino [18]. All putative SNVs were further filtered based on a standard set of criteria to remove the following common types of false calls: (1) the alternative allele was present in the matching normal sample and the contingency between the tumor and normal samples was not statistically significant; (2) the mutant alleles were only present in one stand and the strand bias was statistically significant; (3) the putative mutation occurred at a site with systematically reduced base quality scores; (4) the reads harboring the mutant allele were associated with poor mapping quality. Ambiguous calls were manually inspected to ensure accuracy. Putative indels were evaluated by a re-alignment process to filter out potential false calls introduced by unapparent germline events, mapping artifacts and homopolymer. All mutations were annotated using ANNOVAR [19] with NCBI RefSeq database.

### Detection of somatic sequence mutations in xenograft tumors

To filter out reads caused by mouse stromal contamination in PDX, all reads from the PDXs were run through an *in silico* approach to determine the species of origin. More specifically, we first created a combined reference sequence containing the sequences of all chromosomes in the NCBI genome assemblies of human (GRCh37) and mouse (GRCm38), and then aligned reads from PDX to the combined reference sequence using BWA. Only reads classified of human but not mouse origin were kept in downstream analyses. Afterwards, standard somatic mutation calling was performed on PDX with the matched normal as described previously. For testing purpose, we also performed somatic mutation calling on the uncleaned PDX data to evaluate the effect of mouse contamination on the somatic mutation calling.

### Comparing mutations in xenograft and primary tumors

All unique somatic mutations identified from the primary tumor or PDX were re-visited in both the primary tumor and matched PDX BAM files. The number of mutant and non-mutant reads at the site of each mutation in all BAM files were extracted using Mutation Reads Extractor (manuscript in submission) to calculate coverage and variant allele fraction (VAF).

### Sanger validation

PCR amplicons targeting the PIK3CA and CASP8 regions were generated with gene specific primers (Table S3 and S4) using a touchdown PCR protocol with the following parameters: 94°C for 15 min, followed by 45 cycles of 94°C for 20 seconds, 68°C initial annealing for 30 seconds (followed by 1°C reduction of temperate per cycle to a final annealing temperature of 58°C for remaining 35 cycles), and 72°C for 1 min. Amplicons were purified using the QIAquick^®^ PCR Purification Kit (QIAGEN Inc., Valencia, CA) as per manufacturer instructions. Ten microliter aliquots for each sample were run on a 2% agarose gel for 1 hour at 100V to confirm the correct amplified length (~250 bp). The products were tagged using Big Dye Terminator v3.1 Master Mix Kit (Life Technologies™, Carlsbad, CA) as per manufacturer instructions, and purified over hydrated Sephadex-G50 from Sigma-Aldrich (Sigma-Aldrich^®^, St. Louis, MO), in Multiscreen HV Plates (Thermo Fisher Scientific Inc., Waltham, MA). The eluted samples were placed on a 3130xl ABI Prism Genetic Analyzer and run on the default settings for 50 cm Array using POP-7 Polymer. The data was analyzed with Sequencing Analysis 5.2 software (Life Technologies™, Carlsbad, CA).

### RNA-Seq analysis

Raw reads that passed quality filter from Illumina RTA were first pre-processed by using 1) FASTQC for sequencing base quality control and 2) cutadapt to remove adapter sequences if applicable. Those reads were then mapped to the latest mouse reference genome (mm10) and ENSEMBLE annotation database using Tophat [20] or STAR [21]. A second round of QC using RSeQC [22] was applied to mapped bam files to identify potential RNA-Seq library preparation problems. From the mapping results, reads that matched a single unique location in the genome were kept, allowing up to two mismatches for further analysis. The number of reads aligning to each gene were calculated using HTSeq [23]. Differentially expressed genes were identified using DESeq2 [24], a variance-analysis package developed to infer the statically significant difference in RNASeq data. A biological hypothesis was also tested using a generalized linear model implemented in DESeq2 by construct corresponding contrasts. Multiple testing corrections were applied. The list of differentially expressed genes (DEGs) were analyzed for enriched Gene Ontology and/or KEGG pathway term with the GAGE [25] Bioconductor package. GSAASeqSP [26]was also applied for pathway analysis that utilizes p-values from all genes instead of only DEGs.

### Modelling of mutant structures and affinity binding to PI3K

The experimental structures for mutants BLCAb001 and BLCAb002 Phosphoinositide 3-kinase (PI3K) were not available and hence the mutant structures were modelled using the MODELLER 9.16 [27] using the the crystal structure of Pi3K alpha lipid kinase (PDB ID: 4YKN as template [28]. The constructed models were validated through PROCHECK [29]and ProSA [30]servers. The ligand structure (LY-3023414) was retrieved from the TOXNET's Chem-IDplus database [31]. Sybyl-X 2.0 (Tripos international, USA) [32] was used for the present study.

### Cell cuture and cisplatin resistant cells

BLCAb001 and BLCAb002 cells were isolated from primary tumors of urothelial cell carcinoma of bladder and were originally authenticated by chromosome karyotyping [15]. Cells were cultured using enriched F-medium supplemented with ROCK inhibitor and insulin growth factor as described previously [33]. These cells were recently (< 6 months) confirmed to be of human origin with the detection of the human specific *Alu* gene by RT-PCR. Human bladder cancer cells lines, T24 and UMUC3, were purchased from ATCC. No authentication was done. The cells were cultured in RPMI-1640 medium with 10% FBS. Cisplatin resistant (> 10 fold) cells, T24-Cis and UMUC3-Cis, were generated by continuous treatment of cisplatin (gradual increase) for approximately 5-months.

### Tumor cell growth inhibition

Tumor cell growth inhibitory effect of cisplatin, sulfasalazine alone and in combination, was evaluated by SRB assay [34]. Effect of PI3K/mTOR/AKT inhibitors, LY414, RAD001, and MK2206, was determined by MTT cell proliferation assay kit as described by the manufacturer (ThermoFisher Scientific). LY414 was kindly provided by Eli Lilly and Company. The other compounds were purchased (Selleckchem, Houston TX).

### Colony formation assay

Bladder cancer cells (3 × 10^2^) of parental and cisplatin resistant T24 and UMUC3 cell lines were seeded in plates and allowed to grow overnight. Cells were treated with cisplatin, xCT inhibitor sulfasalazine alone and in combination with cisplatin for 24 to 48 h. Medium was removed and the cells were rinsed with PBS, fresh medium was added, and cells were allowed to grow for 3 to 4 weeks. Cells were then fixed and stained with methylene blue, photomicrographs were captured, and colonies were counted.

### Western blot analysis

Protein extracts were isolated from tumor tissue using a polytron homogenizer and cells were extracted by sonication in lysis buffer. Forty micrograms of protein were separated by gel electrophoresis followed by transfer on to a PVDF membrane. Western blot analysis was performed using the primary antibodies for xCT (1:500 dilution, Abcam), mTOR (1:1000 dilution, Cell Signaling), p-mTOR (1:1000 dilution, Cell Signaling); Akt (1:1000 dilution, Cell Signaling) p-Akt (1:1000 dilution, Cell Signaling), p-PRAS40 (1:1000 dilution) p-S6 (1:1000 dilution, Cell Signaling), β-actin or GAPDH were used as loading controls.

### Quantitative RT-PCR

Gene expression at the mRNA level was determined by performing quantitative RT-PCR (qRT-PCR) as described earlier [34]. Briefly, RNA was isolated from tumor tissue and cells using Trizol reagent and prepared cDNA using the high efficiency cDNA synthesis kit (Life Technologies). Gene specific primers were utilized to determine expression levels of genes with SYBR Green PCR Master Mix (Life Technologies) with CFX96 Touch Real-Time PCR Detection system (Bio-Rad). β-actin was used as an internal control. Normalized fold expression of genes was determined using the CFX Manager Software.

### Patient derived tumor xenografts, drug treatment, and tumor growth

Two patient derived tumor xenografts (PDXs), BLCAb001 and BLCAb002, were generated using fresh primary tumors from bladder cancer patients as described by us earlier [15]. For evaluation of drug efficacy, small pieces of PDXs were surgically transplanted into SCID mice subcutaneously and allowed to establish. When the tumors reached approximately 100-200 mm^3^, mice were randomized into groups of 5-8 mice and treated with vehicle, LY414 (5 mg/kg BID by oral gavage), or cisplatin 5 mg/kg or 10 mg/kg weekly by intraperitoneal injection). Tumor sizes were blindly measured weekly along with the body weight of mice. At the end of the treatment, tumors were collected, weighed, and processed for formalin fixation and small pieces were frozen for protein extraction.

### Immunohistochemical evaluation

We determined the expression of various molecular markers in the original cystectomy tumor samples and their PDXs BLCAb001 and BLCAb002 along with human bladder cancer tumors arranged in tissue microarray (TMA). Standard immunohistochemical protocols were followed as previously described by us earlier [15, 35]. Briefly, formalin fixed paraffin embedded tumors were used to prepare sections (5 μm), deparaffinized followed by rehydration and antigen unmasking in sodium citrate buffer (pH 6.0). Primary antibodies xCT (1:400 dilution), AKT, p-AKT, mTOR, p-mTOR, and cytokeratin 5/6/20, were used for overnight incubation at 4°C, followed by their respective horseradish-conjugated secondary antibody for 1hr. Photomicrographs were captured using a Zeiss Axio (Peabody, MA) microscope.

## SUPPLEMENTARY MATERIALS TABLES AND FIGURES




