# Supplementary material for: Genomic profiling is predictive of response to cisplatin treatment but not to PI3K inhibition in bladder cancer patient-derived xenografts
Source: Oncotarget. 2016 Nov 3;7(47):76374–89. doi: 10.18632/oncotarget.13062 (PMC5363516; doi:10.18632/oncotarget.13062)
Supplement: Supplementary file 1 [file oncotarget-07-76374-s001.pdf]

Genomic profiling is predictive of response to cisplatin treatment but not to PI3K inhibition in bladder cancer patient-derived xenografts

Supplementary Material

Table S1. Statistics about whole-exome sequencing (WES) of human and xenograft samples.

Table S2. Point mutations identified in primary tumor or PDX by WES.

Table S3. Sanger confirmation of PIK3CA and CASP8 mutations.

Table S4. Gene specific primers for PCR amplicons targeting the PIK3C and CASP8 regions.

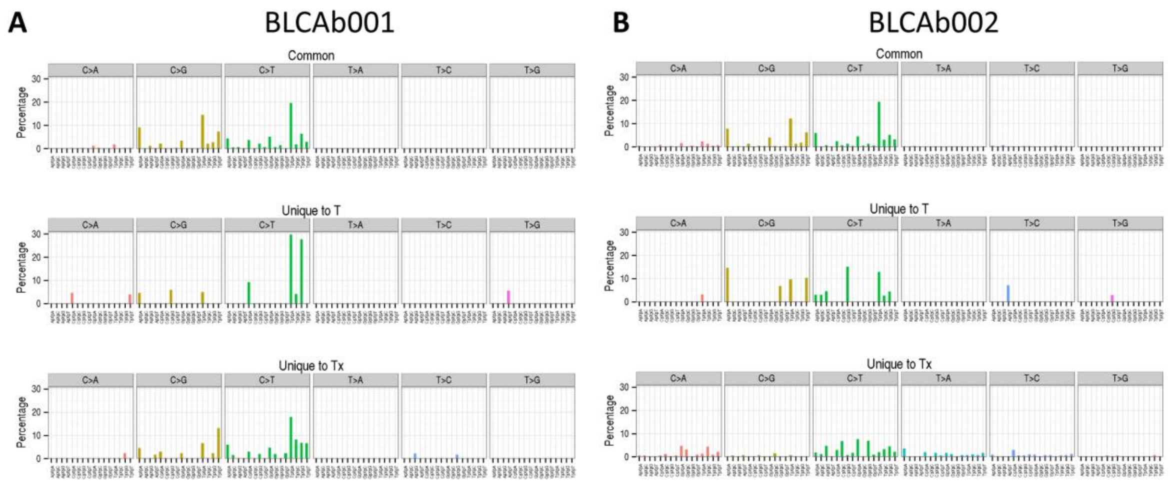

Figure S1. Mutational signature in primary tumors and BLCAb001 and BLCAb002. (A) BLCAb001 and (B) BLCAb002 mutational signature: common (upper), tumor (middle) and PDX (lower).

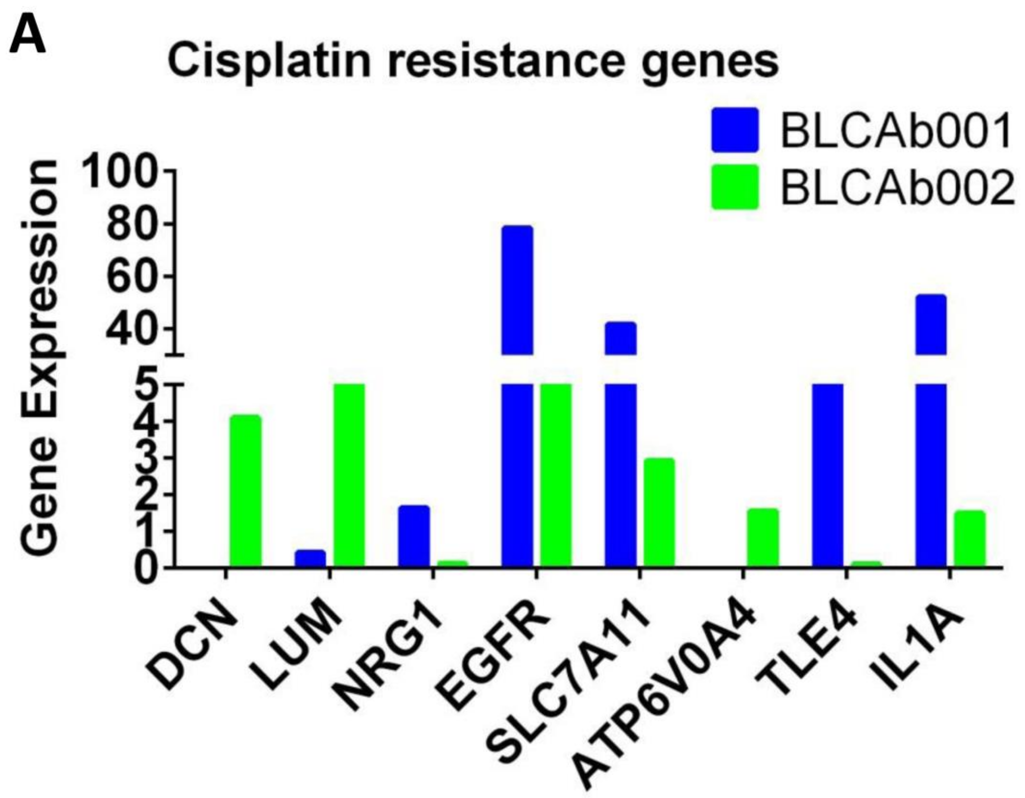

Figure S2. Differential expression of cisplatin associated genes in BLCAb001 and BLCAb002. RNA-eq analysis revealed differential expression of genes associated with cisplatin resistance in BLCAb001 and BLCAb002 PDXs.

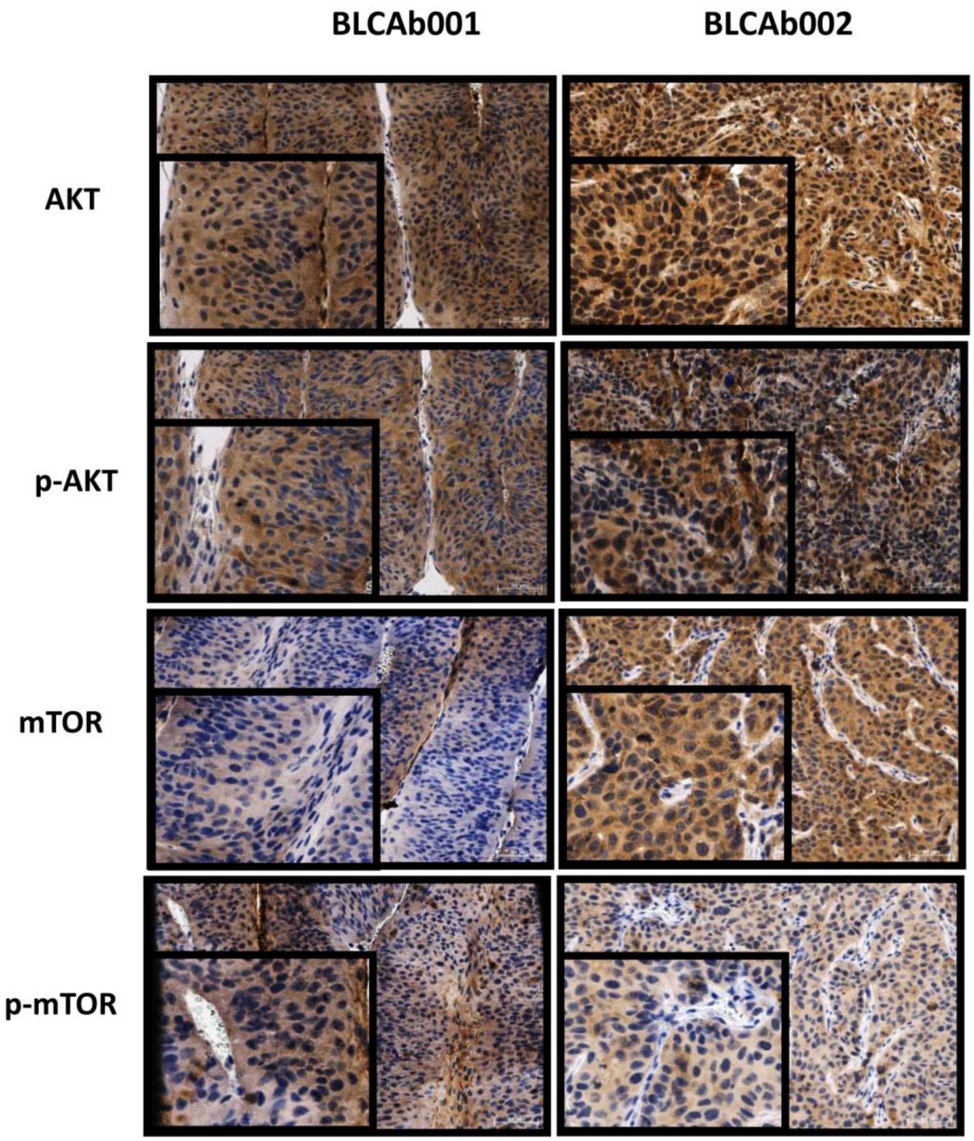

Figure S3. Immunohistochemical characterization of BLCAb001 and BLCAb002. Representative pictures of immunostaining for AKT, p-AKT, mTOR, p-mTOR in BLCAb001 and BLCAb002 are depicted.

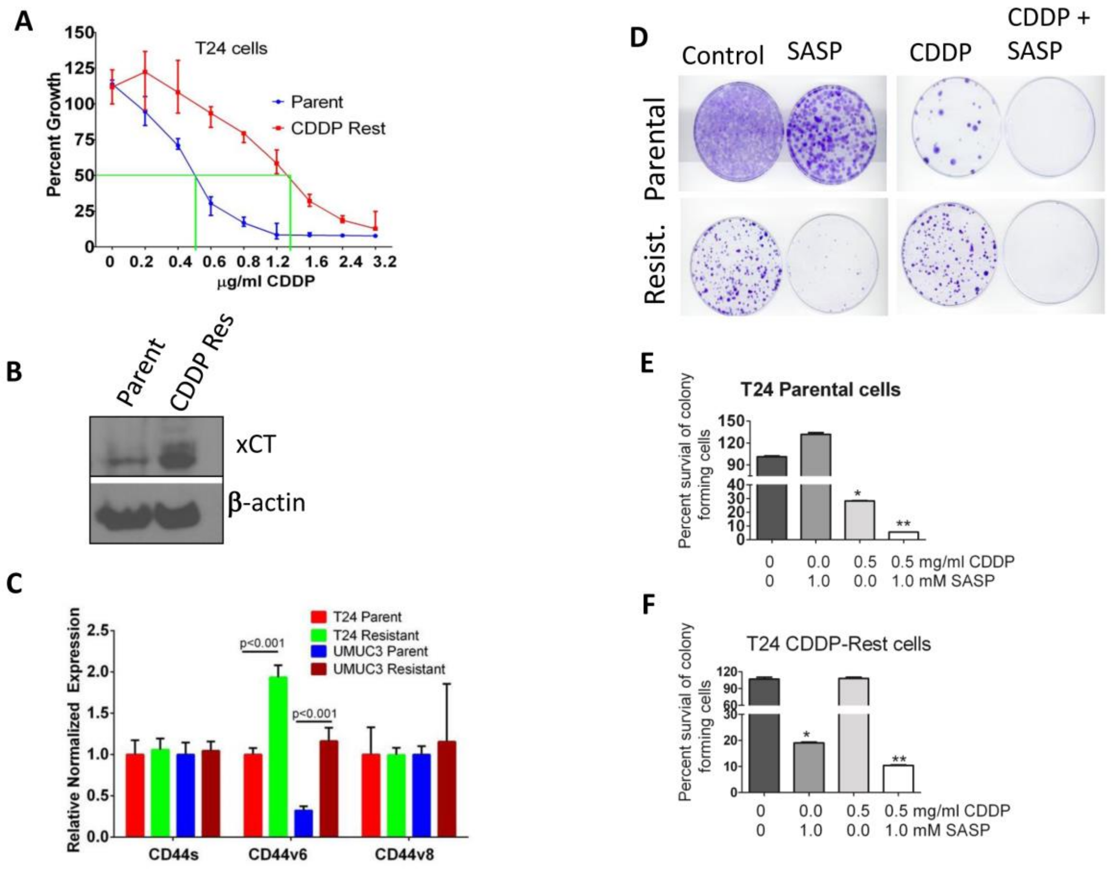

Figure S4. Effect of xCT inhibition on cytotoxic effects of cisplatin. (A) Generation of cisplatin resistant T24 cells with repeated exposure to cisplatin. (B) Western blot analysis shows the upregulation of xCT in T24 cisplatin resistant cells as compared to parental cells. (C) QRT-PCR analysis of CD44 standard and variant forms v6 and v8 in parental and cisplatin resistant T24 and UMUC3 cells. (D) Colony formation assay of parental and cisplatin resistant T24 cells treated with cisplatin (CDDP), SASP alone and in combination. (E-F) Means percent ± SE survival of colony formation in T24 parental and cisplatin resistant cells are reported; * $p \leq 0.001$ vs. control: ** $p \leq 0.0001$ vs. single agents.

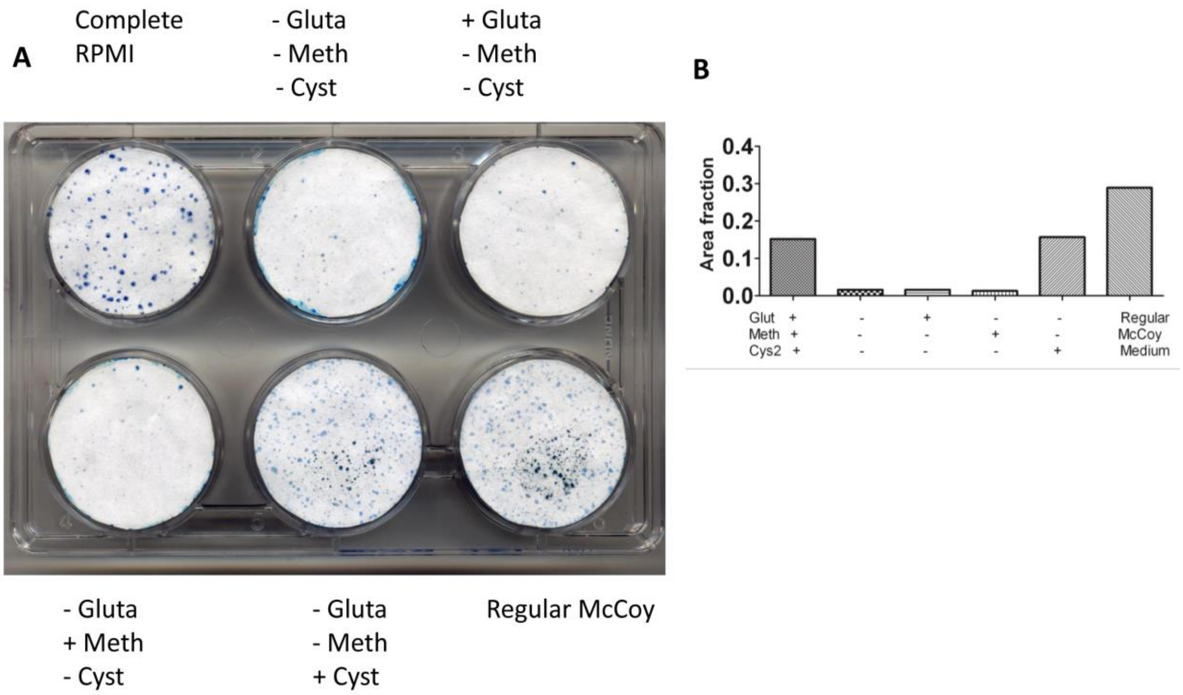

Figure S5. Cystine supplementation rescues T24 cells growth in depleted media. (A) T24 cells colony formation in complete RPMI media and special medium with the depletion of glutamate, methionine, cystine and supplementation of individual amino acids separately. Regular McCoy medium was used as control medium to compare the growth. (B) Image J quantification of colony forming area in cells cultured with and without specific amino acids.

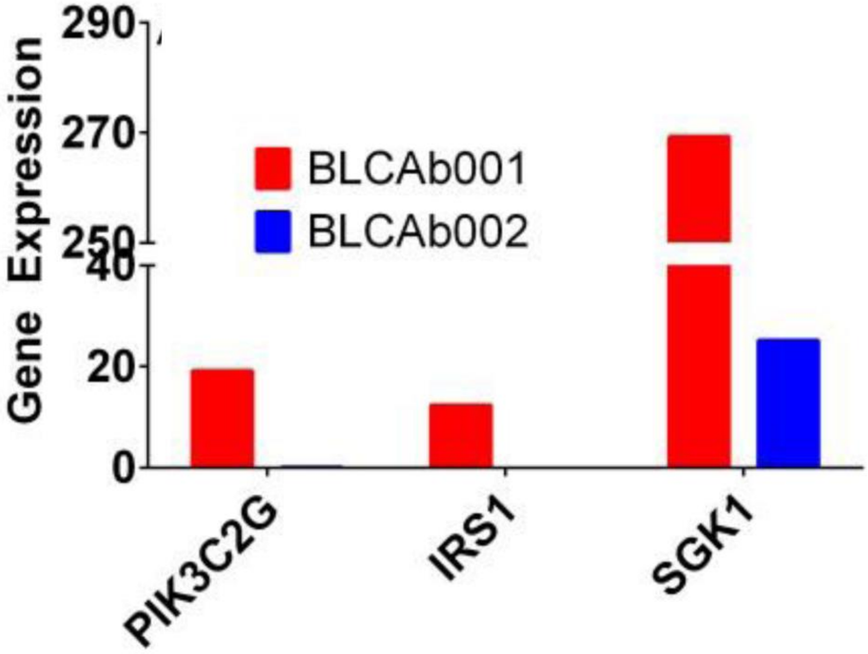

Figure S6. **Differential expression of genes associated with resistance to PI3K/AKT inhibitors**. Increased expression of genes in BLCAB001 tumors. RNA-eq data analysis showing the differential expression of PI3K/AKT inhibitor resistance genes in BLCAb001, as compared to BLCAb002.

A

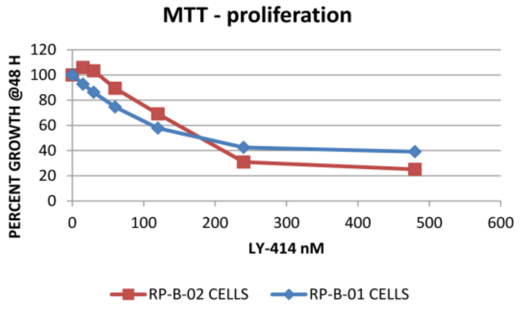

B

	m	Dm	r
BLCAb001	0.909	212.9	0.98
BLCAb002	1.89	208.8	0.97

Figure S7. Effect of LY414 on BLCAb001 and BLCAb002 in enriched F medium. (A) MTT assay - Tumor cell growth inhibitory effect of LY414 on cells in enriched F medium. (B). Concentration required to inhibit 50% of growth (IC50) or Dm (median effect dose) of LY414 in F medium, r is correlation coefficient, quantifies the direction and magnitude of correlation. IC50 values calculated using the GraphPad Prism software (San Diego, CA).

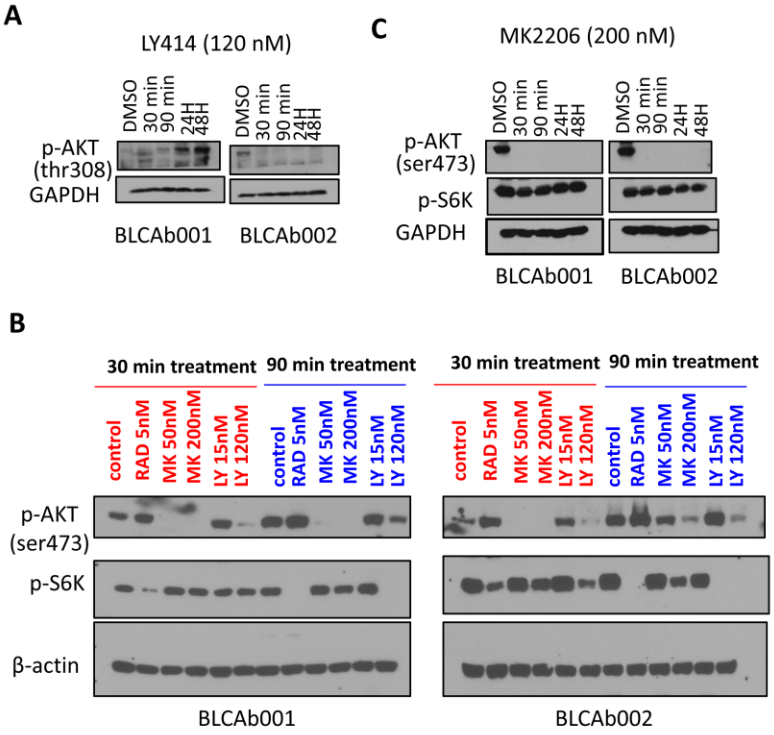

Figure S8. Effect of PI3K/AKT/mTOR inhibitors on BLCAb001 and BLCAb002 cells. (A) BLCAb001 and BLCAb002 cells were treated with MK2206 (AKT inhibitor), RAD001 (mTOR inhibitor) and LY414 (PI3K/mTOR inhibitor) for 30-90 minutes in DMEM. Western blot analysis shows the treatment effect on p-AKT and p-S6K in BLCAb001 and BLCAb002 cells. (B) BLCAb001 and BLCAb002 cells were treated with MK2206 for 30, 90 minutes, 24 and 48 hrs in DMEM. Western blot analysis shows the treatment effect on p-AKT and p-S6K in BLCAb001 and BLCAb002 cells. (C) LY414 treatment effects on p-AKT (T308) isoform in BLCAb001 and BLCAb002 cells.

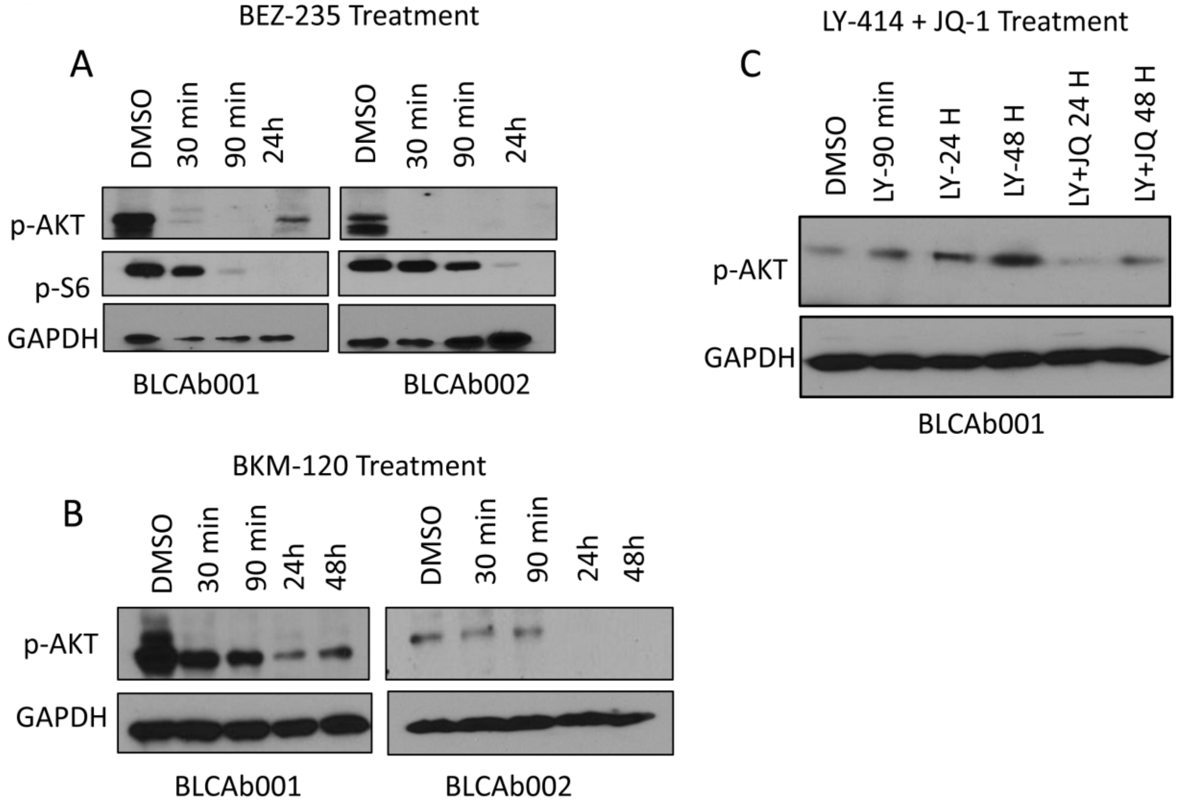

Figure S9. Effect of BEZ 235 (PI3K/mTOR inhibitor), BKM120 (PI3K inhibitor), and combination of LY414 (P13K/mTOR inhibitor) with JQ-1 (bromodomain inhibitor) on p-AKT and p-S6 in BLCAb001 and BLCAb002 cells. (A) Cells treated with 250nM of BEZ-235 for 30, 90 min and 24h. Western blot analysis revealed that p-AKT was inhibited at early hours in BLCAb002 then it was found rebounded at 24h. Rebound was not observed in BLCAb002 cells. (B) BKM 120 (500nM) was used to treat the cells for 30min, 90 min, 24h and 48h and pAKT levels were detected by western blot analysis. Inhibition of p-AKT was found to be more pronounced in BLCAb002 cells compared to BLCAb001 cells. (C). BLCAb001 cells were treated with LY414 alone and in combination of JQ1 and determined p-AKT levels after 90 min, 24h , and 48h. Rebound p-AKT was found to be inhibited with the combination of JQ-1.

A

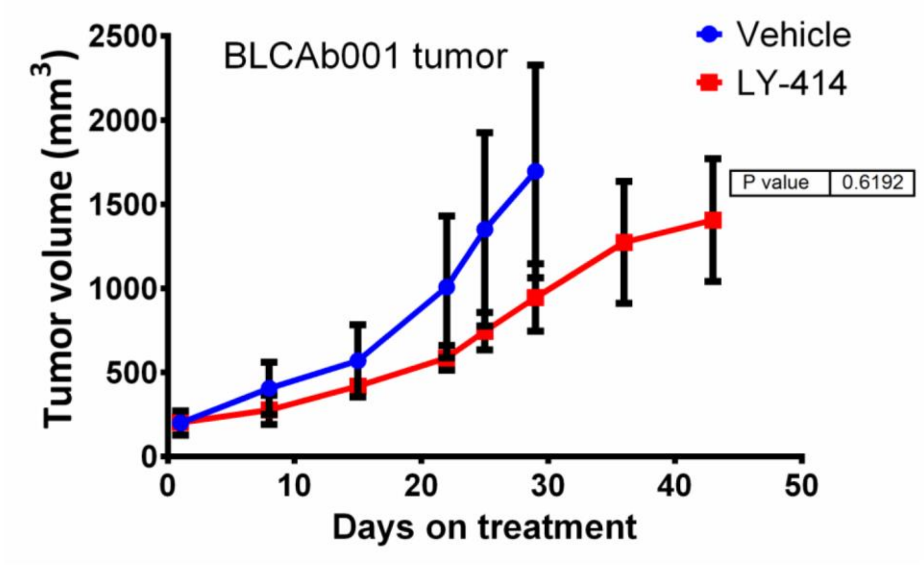

B

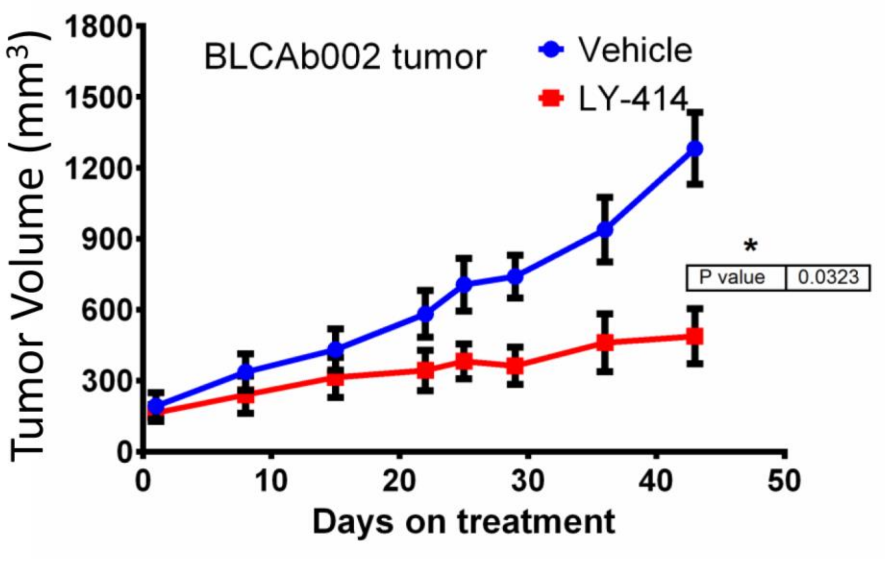

Figure S10. Tumor growth inhibition was found significant in BLCAb002 tumors compared to BLCAb001 tumors. BLCAb001 and BLCAb002 tumors were implanted in SCID mice and allowed to establish. When both the tumors grow and reach about 200mm3 size, LY414 drug treatment was initiated and continued for 45 days. Tumor size was measured weekly,

(A) BLCAB001 tumors (n =4) were treated with LY414. No significant tumor growth inhibition ($p < 0.619$) by LY treatment in BLCAb001 tumors. (B) BLCAb002 tumors (n=4) were transplanted in SCID mice and treated with LY414. Significant tumor growth inhibition ($p<0.0323$) was observed with LY414 treatment.
